# Experiences of Decision-Making in Healthcare and Online Health Information-Seeking Among Older Adults and People with Long-Term Disease: Online Survey Study

**DOI:** 10.1177/23743735251415086

**Published:** 2026-01-20

**Authors:** Milla Rosenlund, Tuuli Turja, Virpi Jylhä, Kaija Saranto, Hanna Kuusisto

**Affiliations:** 1 Department of Health and Social Management, 4344 University of Eastern Finland, Kuopio, Finland; 2 Faculty of Social Sciences, 196418 Tampere University, Tampere, Finland; 3 Research Centre for Nursing Science and Social and Health Management, 60650 Kuopio University Hospital, Kuopio, Finland; 4 Department of Neurology, 60670 Tampere University Hospital, Tampere, Finland

**Keywords:** shared decision-making, patient participation, patient communication, health information

## Abstract

Healthcare decision-making (DM) has shifted from a paternalistic model to shared DM, where professionals contribute with clinical expertise and patients share their values and preferences. Simultaneously, access to online health information influences how patients engage in decisions concerning care. This study examined perceived DM experiences during doctor's appointments and online health information-seeking among older adults and individuals with long-term conditions. A total of 736 Finnish respondents (mean age 68 years) completed an online survey. The Shared Decision-Making Questionnaire (SDM-Q-9-FIN) assessed involvement in clinical decisions. Most participants reported feeling involved, with a mean SDM-Q-9 score of 25.96/36. Longer appointment duration (β = .50, *P* < .001), higher education attainment, and better health status were positively associated with perceived involvement. Adherence to treatment also enhanced DM experiences. Nearly half (48.6%) did not seek online health information before appointments. Respondents from patient associations reported more frequent information-seeking. The findings suggest that adequate consultation time and tailored communication can enhance DM. Support should be prioritized for patients with lower health status or limited health literacy.

## Introduction

Decision-making (DM) in health policy and clinical practice has shifted from a paternalistic model—where healthcare professionals (HCPs) make decisions independently—toward patient-centered shared decision-making (SDM).^1,2^ Unlike informed DM, where patients decide based on information provided by HCPs,^2,3^ SDM emphasizes collaboration, integrating clinical expertise with patients’ values.^4^ SDM is a structured process in which HCPs contribute medical knowledge while patients are encouraged to express their preferences and needs.^5–9^ This development reflects a broader paradigm shift toward participatory and patient-centered care.^10,11^ SDM is particularly beneficial in preference-sensitive conditions such as multiple sclerosis (MS) and epilepsy, where different treatment options exist.^12,13^ Furthermore, patient-centered care aligns with person-centered care, recognizing the patient as a whole person beyond their diagnosis.^14^

SDM has many positive implications as it promotes patient autonomy emphasizing the patient's right to make treatment decisions based on available options,^6,15^ enhancing engagement in health management and reducing DM conflicts.^16^ Furthermore, SDM increases patient satisfaction,^17^ self-efficacy,^16^ and health outcomes especially in disadvantaged population groups.^15,16^ However, successful SDM depends on various factors, including healthcare system structures and appointment characteristics (such as interaction and whether the consultation is offline or online), as well as patient-related aspects such as the patient's background, their interaction with HCPs, and the nature of the decision.^18–20^

Not all patients are ready to participate in SDM; readiness is shaped by prior knowledge, attitudes, health literacy, and evaluative skills.^21^ Supporting engagement requires attention to individual capabilities and fostering inclusion.^22^ Health literacy is essential for active participation and encompasses the ability to access, understand, evaluate, and communicate health information.^21,23,24^ At the same time, online health information-seeking, shaped by patients’ health literacy, plays an increasing role in the patient-HCP relationship and supports DM.^25–27^

While SDM has demonstrated benefits, further research is needed to assess current practices and the impact of sociodemographic and contextual factors.^15–17^ This study examined experiences of SDM during doctor's appointments using the Finnish version of the 9-Item Shared Decision-Making Questionnaire (SDM-Q-9-FIN)^28^ focusing on the role of sociodemographic background and health information-seeking behavior.

We proposed the following research question:

How do older adults and people with long-term disease experience DM at the doctor's appointment, and how is the experience influenced by sociodemographic factors and health information-seeking behavior?

## Methods

The data were collected with an online survey conducted between December 2021 and January 2022. Members of the Finnish Pensioners’ Federation (ca. 120 000 members) and the Finnish Neuro Society (ca. 10 000 members) were targeted due to their characteristics (older adults and respondents with long-term disease). The Finnish Pensioners’ Federation is a federation aimed at pensioners and older adults. In Finland, the retirement age is currently around 63-65 years but is gradually increasing. The questionnaire was also shared with the Finnish Epilepsy Association (ca. 10 000 members) and the Organization for Respiratory Health in Finland (ca. 25 000 members). The Finnish Pensioners’ Federation sent the invitation to participate via its member database e-mails (N = 30329). All the organizations shared a link to the survey on their social media accounts (Facebook and Twitter) and their official website. The Finnish Neuro Society additionally shared the link in their newsletter. Respondents were permitted to respond only once, as enforced by the Webropol survey settings. In the analysis, the Finnish Pensioners’ Federation was considered one group and the respondents from the patient associations ((ie, the Finnish Neuro Society, the Finnish Epilepsy Association and the Organization for Respiratory Health in Finland) a single group because of low individual numbers of responses. In this study, we used a sub-sample (n = 736) extracted from the total cohort (N = 1935). The sub-sample consisted of respondents who reported having attended a doctor's appointment within the previous 6 weeks where a treatment decision was made. Respondents did not receive compensation for participating in the survey.

The study followed guidelines and regulations. It was approved by the Ethics Committee of the Tampere Region, Finland (ID 18/2021) and complied with the Finnish Advisory Board of Research Integrity regulations and the World Medical Association Declaration of Helsinki. The respondents were informed about the study, the use of the data, data protection, storage, and rights to use the data. Before beginning the questionnaire, respondents were asked for their consent to participate in the research. The respondents’ anonymity, confidentiality, and informed consent were maintained while gathering and handling the data. No direct identifiers of respondents were gathered in the survey.

### Survey Items

In the survey the respondents were asked about their sociodemographic background and subjective health status. The primary content of the survey was the Finnish version of the SDM-Q-9 questionnaire, SDM-Q-9-FINwhich measures the self-reported experience of DM in healthcare.^28,29^ Respondents were able to complete the SDM-Q-9-FIN only if they reported having had a doctor's appointment where a treatment decision was made within the past 6 weeks.^30^ Health information-seeking behavior which included understanding and using health information were inquired after as well. The survey items are listed in Appendix 1 and Appendix 2.

#### Respondent Characteristics and Perceived Experiences of Decision-Making

The sociodemographic information of the respondents collected in the survey included age, gender, and education. Respondents also self-assessed their current health status using a 5-point Likert scale ranging from good (1) to poor (5).^31^ Prior to completing the SDM-Q-9-FIN, respondents were asked to reflect on their most recent appointment. Respondents evaluated whether the appointment duration was sufficient to discuss all relevant issues, using a 4-point Likert scale from completely disagree (1) to completely agree (4).^32,33^

The perceived experience of SDM during the appointment was assessed using the SDM-Q-9-FIN.^28^ Originally developed in German to measure patient involvement in healthcare DM, permission for translation and use was obtained from the core development team at the University Medical Center Hamburg-Eppendorf.^29^ The nine items were answered on a 4-point Likert scale (completely disagree = 1 to completely agree = 4), yielding a total score range of 9 to 36.

#### Online Health Information-Seeking Behavior

Online health information-seeking behavior was assessed by asking, “How often do you search for information concerning your own health?”^34^ The item was answered on a 7-point Likert scale ranging from “not at all” (=1) to “daily” (=7). The respondents were also asked whether “The diagnosis or treatment decision made by the doctor contradicted the information I had found beforehand on the Internet” and “The diagnosis or treatment decision I received contradicted my own feelings.”^35–37^.

Regarding understanding health information respondents were shown two statements: “I have enough information to participate in a discussion about my health and I understand what the findings concerning my treatment mean.” The statements were applied from the eHealth Literacy Questionnaire questionnaire.^38^ All statements were answered on a 4-point scale ranging from “completely disagree” (= 1) to “completely agree” (= 4).

### Data Analysis

Analysis was first conducted by describing the frequency, percentage, mean, and standard deviation (SD) for each item and the total score of the SDM-Q-9-FIN. The reliability of the Finnish version of the SDM-Q-9 was measured using Cronbach's alpha; Cronbach's alpha was 0.92, which was in line with previous studies.^29,39–46^ Confirmatory factor analysis (CFA) was used to verify the dimensionality and structural validity of the questionnaire. The validation of the Finnish version of the SDM-Q-9 is reported elsewhere.^28^ Descriptive statistics (frequencies, percentages) were used to describe the characteristics of the respondents, doctor's appointments, and various factors. Statistical significance tests (chi-square test, Mann-Whitney U test, or Kruskal-Wallis test) were used to describe differences between the respondent groups.

Based on the results of the CFA, the SDM-Q-9-FIN items were treated as a single sum variable, which served as the dependent variable in modeling the association between sociodemographic and other contextual factors with perceived DM. The results of linear regression analysis were reported as explanatory power (*R*^2^), standardized regression coefficients (*β*), and statistical significance (*P*). *P*-values of <.05 were considered significant. Analysis of variance (ANOVA) for regression was reported to measure the fitness of the linear regression model. Descriptive statistics and other statistical analyses were performed using SPSS version 29.0.^47^ CHERRIES (Checklist for Reporting Results of Internet E-Surveys) checklist was used to guide the reporting of the survey^48^ (Appendix 3).

## Results

### Characteristics of the Respondents and the Doctor's Appointment

A total of 736 respondents who had had a doctor's appointment during the previous 6 weeks answered the questionnaire. Respondents’ sociodemographic background (gender, age, and education), their subjective health status, and appointment-related characteristics are described in Table 1.

**Table 1.** Sociodemographic Background of the Respondents (N = 736) and Appointment-Related Characteristics.

Count n (%)	Finnish Pensioners’ Federation 629 (85.4%)	Patient associations* 107 (14.5%)	Of the total (N = 736)
*Sociodemographic factors and subjective health status*			
**Gender**			
Women	378 (60.1%)	94 (87.9%)	472 (64.1%)
Men	251 (39.9%)	13 (12.1%)	264 (35.9%)
Other	0	0	0
**Age**			
Mean (years)	71 yrs	52 yrs	68 yrs
**Highest education level**			
Elementary school or similar	88 (14.0%)	4 (3.7%)	92 (12.5%)
High school or vocational education	164 (26.1%)	39 (36.4%)	203 (27.6%)
Bachelor's degree	273 (43.4%)	34 (31.8%)	307 (41.7%)
Master's degree	94 (14.9%)	25 (23.4%)	119 (16.2%)
Doctoral or licentiate degree	10 (1.6%)	5 (4.7%)	15 (2.0%)
**Subjective health status**			
Good	95 (15.1%)	3 (2.8%)	98 (13.3%)
Fairly good	259 (41.2%)	25 (23.4%)	284 (38.6%)
Average	216 (34.3%)	43 (40.2%)	259 (35.2%)
Fairly poor	59 (9.4%)	30 (28.0%)	89 (12.1%)
Poor	0	6 (5.6%)	6 (0.8%)
*Appointment characteristics*			
**Reason for the appointment**			
Long-term condition (eg, medication, follow-up)	246 (39.1%)	69 (64.5%)	315 (42.8%)
Diagnosing the symptom/disease	360 (57.2%)	35 (32.7%)	395 (53.6%)
Preventive care	21 (3.3%)	3 (2.8%)	24 (3.3%)
Other reason	1 (<1%/0.4%)	1 (<1%/0.9%)	2 (<1%/0.3)
**Purpose of the appointment**			
First appointment	44% (273)	26% (28)	41% (301)
Follow-up	33% (207)	30% (32)	32% (239)
Periodic follow-up for long-term illness	24% (148)	44% (47)	26% (195)
Not known			1% (1)
**Did you visit at…**			
General practitioner	52% (328)	23% (25)	48% (353)
Specialist	66% (70)	66% (70)	49% (363)
Occupational physician	11% (12)	11% (12)	3% (20)
**Location of the appointment**			
Face to face	93% (587)	79% (84)	91% (671)
Phone call	6% (40)	19% (20)	8% (60)
Video or other distance connection	<1% (2)	2% (3)	< 1% (5)
**The appointment duration was sufficiently long enough to discuss all the relevant issues**			
Completely disagree	34 (5.4%)	15 (14.0%)	49 (6.7%)
Partly disagree	65 (10.3%)	11 (10.3%)	75 (10.2%)
Partly agree	123 (19.6%)	35 (32.7%)	158 (21.5%)
Completely agree	407 (64.7%)	46 (43.0%)	453 (61.6%)

Despite the COVID-19 pandemic occurring during the time of the survey (December 2021-January 2022), nearly all appointments, 671 out of 736 (91.1%), took place in person with either general practitioner (n = 353, 47.9%) or a specialist (n = 363, 49.3%).

Most of the respondents felt that the appointment duration was sufficiently long to discuss all the relevant issues, although 26 out of 107 (24.3%) respondents from patient associations disagreed either partly or completely with *X^2^* (6, N = 736) = 32.32, *P* < .001.

### Perceived Experience in Decision-Making

In the total sample, the mean score (SD) for perceived experience was 25.96 (SD 7.59). Respondents from the Finnish Pensioners’ Federation had scores above the mean of 26.38 (SD 7.56), contrary to that of respondents from the patient associations (24.14, SD 6.92). Table 2 presents the average total scores among different respondent groups, gender, education level and statistical significance between the groups.

**Table 2.** Variation of Perceived Experience of DM in Sociodemographic Groups.

Respondent group	SDM mean (SD)	Difference	*P-*value
		−3.861^a^	.001
Finnish Pensioners’ Federation	26.38 (7.56)		
Patient associations	24.14 (6.92)		
Total	25.96 (7.59)		
**Gender**		−.611^a^	.541
Women	25.78 (7.80)		
Men	26.27 (7.19)		
**Education**		7.692^b^	.104
Elementary school or similar	26.84 (7.51)		
High school or vocational education	26.15 (7.48)		
Bachelor's degree	26.03 (7.48)		
Master's degree	24.46 (7.68)		
Doctoral or licentiate degree	28.47 (6.38)		

^a^ Mann-Whitney U test; ^b^Kruskal-Wallis test.

DM, decision-making; SDM, shared decision-making; SD, standard deviation.

The mean score for single items ranged between 2.44 (item 7) and 3.38 (item 1), while for all items, it was 2.88. The mean score was slightly higher among the respondents from the Finnish Pensioners’ Foundation (2.93) than among the respondents from patient associations (2.76). The mean score among men (2.92) was slightly higher than for women (2.80). The results for various respondent groups are presented in Appendix 4.

### Online Health Information-Seeking Behavior

Respondents from patient associations and with long-term conditions reported searching for information online more often than respondents from the Finnish Pensioners’ Federation; 28 out of 107 (26.1%) of the respondents from patient associations said that they searched for health information daily, once or a few times a week. Almost half of all respondents, 329 out of 736 (45%), did not search for health-related information at all or did so only a few times a year. The results are presented in more detail in Table 3.

**Table 3.** Variation in Online Health Information-Seeking Behavior Between Respondent Groups.

Item	Response	Finnish Pensioners’ Federation (n = 629)	Patient Associations (n = 107)	Total (N = 736)	*P-value*
**Searching information**	Few times/year	302 (48.0%)	27 (25.2%)	329 (44.7%)	.001
	Monthly	288 (45.8%)	52 (48.6%)	340 (46.2%)	
	Weekly	37 (5.9%)	27 (25.2%)	64 (8.7%)	
	Daily	2 (0.3%)	1 (1.0%)	3 (0.4%)	
**Doctor's decision contradicted the information found beforehand**	Completely or partly disagree	252 (40.1%)	51 (47.7%)	303 (41.2%)	.002
	Completely or partly agree	63 (10.0%)	12 (11.2%)	75 (10.2%)	
	Did not search online	314 (49.9%)	44 (41.1%)	358 (48.6%)	
**Enough information to discuss my health**	Completely or partly disagree	97 (15.4%)	22 (20.6%)	119 (16.2%)	.041
	Completely or partly agree	532 (84.6%)	85 (79.4%)	617 (83.8%)	
**Understand treatment findings**	Completely or partly disagree	76 (12.1%)	20 (18.7%)	96 (12.9%)	.105
	Completely or partly agree	553 (87.9%)	87 (81.3%)	640 (86.9%)	

Many of the respondents, 358 out of 736 (48.6%) answered that they did not search for information before receiving a diagnosis or making a treatment decision. Of those who had sought information before the appointment, 75 out of 736 (10.2%) respondents completely or partly agreed that the diagnosis or treatment contradicted the information they had found themselves.

### Explanatory Factors to Perceived Experiences of Decision-Making

**Figure 1.**
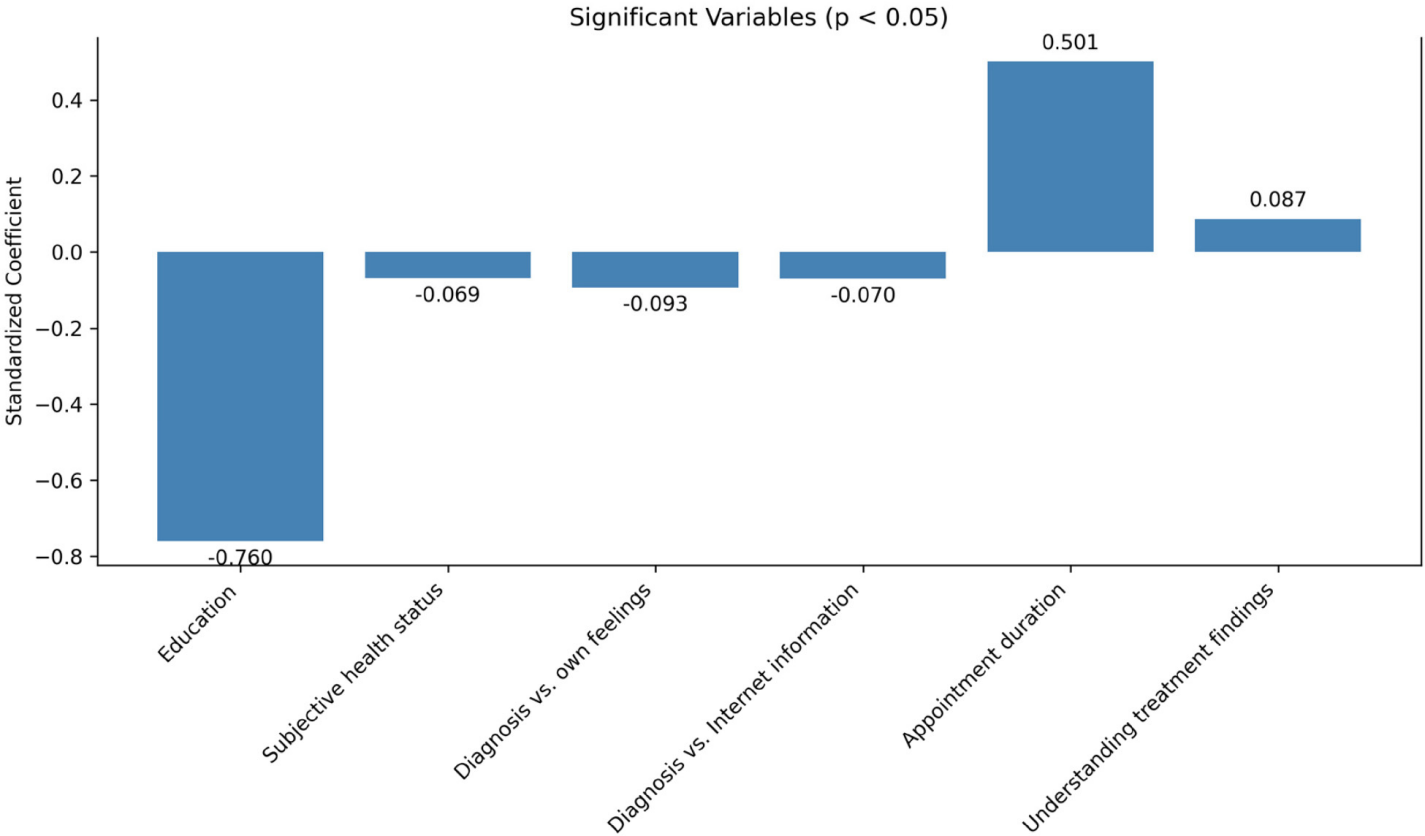
Significant variables contributing to the variance in perceived DM at the doctor’s appointment. DM, decision-making.

Linear regression analysis was used to examine the factors associated with the perceived experience of realized SDM. Results are presented in Table 4 and Figure 1.

**Table 4.** Model of the Variables Correlating with Perceived DM at the Doctor's Appointment.

Independent variable	Standardized regression coefficient (*β*)	Std. Error	*P*-value
Constant (SDM)		1.97	*<*.001
Age	0.029	0.024	.268
Gender	0.027	0.486	.875
Education	−0.76	0.241	.012
Subjective health status	−0.069	0.280	.038
Diagnosis or decision contradicted my own feelings	−0.093	0.249	.004
Diagnosis contradicted the information found in internet	−0.070	0.131	.026
Appointment long enough to discuss the relevant issues	0.501	0.268	*<*.001
Prevalence of searching for health information	0.014	0.215	.673
Having enough information to participate in a discussion about the health	0.047	0.367	.191
Understanding what the findings concerning the treatment mean	0.087	0.384	.018

DM, decision-making; SDM, shared decision-making.

In regression analysis, the variables in the model explained 36.3% of the variance in the perceived experience of DM with an adjusted R2 = .363. The results of ANOVA (F(10, 725) = 42.798, *P* *<* .001) indicate the model's goodness of fit.

Of the independent variables, appointment duration made the most considerable contribution (β = .501) to explain the variation in SDM, when the variance of all the other variables was controlled.

## Discussion

In this study, we measured perceived experience of DM during doctor's appointments, utilizing the SDM-Q-9-FIN questionnaire. We also explored the association between sociodemographic factors (age, education, gender, and subjective health status), other contextual factors, and online health information-seeking behavior with perceived experience of DM. Most respondents reported that key elements of SDM, as measured by the SDM-Q-9-FIN questionnaire, were present during their most recent appointment. These elements include discussing treatment options, weighing benefits and risks, and considering patient preferences. However, respondents from patient associations experienced less involvement in DM during the doctor's appointment compared to the older adults’ group. Studies have shown that SDM would be especially beneficial for patients with preference-sensitive conditions, including neurological conditions such as MS.^49–51^

Appointment duration proved to have the most significant impact on the experience of DM during the doctor's visit. The importance of having a sufficiently long appointment duration to discuss all relevant issues is in line with previous studies, as the length of the consultation has been identified as a significant factor affecting the experience of SDM among older adults.^52^ In addition, SDM has been linked to decreased appointment length.^53,54^

Contradictory information the respondent reported having with regard to the received diagnosis or decision on treatment, also stood out with a significant role in experience of DM. Older adults, as a majority in our data, are known to seek information as frequently as younger adults, but they have lower health literacy scores, when it comes to online information.^55,56^ However, based on prior studies showing no generational differences in seeking online health information,^57^ our study suggests that the contradiction between information sought beforehand and the received medical diagnosis or decision on treatment is a generalizable explanatory factor of perceived experience of DM. When patients find information before a medical appointment that does not align with the received decision, it can make them feel less involved in SDM.^58^ Notably, our study shows that this happens regardless of age and gender.

One of the other most significant explanatory factors referred to health literacy. Respondents who scored high in understanding medical findings reported more probably about the experienced SDM. Health literacy skills may be considered among the useful skills for SDM to support patient empowerment and self-efficacy,^21,23,59^ but the association between information seeking and understanding and SDM is not that apparent. In prior studies, online information-seeking has been found relevant, for example among patients with MS^60^ and epilepsy.^61–63^ Our study showed that over one-quarter of respondents who had sought information before the appointment felt that the chosen treatment was consistent with the information they found online. Another study has shown that information on treatment was one of the most common topics searched for among primary care patients.^64^ However, an interesting finding in our study was that nearly half of the respondents did not search for information online before the doctor's appointment. Among those who had searched for information, it had no association with perceived experience of DM.

Regarding how the background information of the respondents associated with perceived experience of DM, subjective health and education appeared to be significant factors. Respondents in relatively good health reported higher prevalence of SDM, in line with research that has demonstrated a correlation between poor health status and lower engagement in medical DM.^65,66^ One's educational attainment also appeared to play a significant role in the probability of experienced SDM. The impact of a high level of education on perceived SDM varies among studies.^20^ Our study contributes with a finding that a relatively high education still manages to explain some of the variance in perceived experience of DM. However, the remaining question with the explanatory factors is whether respondents with certain profiles are given more opportunities to participate in the DM, or do, they just recognize it is better.

### Strengths and Limitations

A strength of this study is the use of the internationally validated SDM-Q-9 questionnaire, which has consistently demonstrated reliability across diverse patient populations. The instrument offers a concise and coherent measure of patient-reported SDM experiences. The study adds to the limited Finnish literature by exploring SDM among older adults and individuals with chronic conditions in outpatient settings, particularly in the context of digital health advancements and post-pandemic care.

Several limitations relate to our study. First, the SDM-Q-9 is only one instrument for measuring the perceived experience of DM in healthcare. Second, the vast majority of the respondents (85%) were elderly people which limits the generalizability of results to younger populations. Because of the imbalance among the sub-samples, only 15% of the respondents had long-term conditions and most respondents rated their subjective health status as good or average. Including more participants with long-term conditions and poorer self-reported health status would enhance the representativeness of the sample and improve the generalizability of the results. Another limitation regarding the respondents is potential sampling bias, as those recruited through patient federations or societies may be more engaged in the society, as well as their care, compared to the broader patient population. Thirdly, the survey was sent electronically, which may have affected the response rate for people lacking digital device skills. Additionally, many other factors may affect DM in healthcare. Only a few of them were investigated in this study. Finally, it is worth considering that the study was conducted in Finland, where the healthcare system is different from that in many other countries. This affects the global generalizability of the findings to other countries.

## Conclusions

This study explored how older adults and individuals with long-term conditions experience DM during doctor's appointments, and how the experience is influenced by sociodemographic factors and health information-seeking behaviors. The majority of the respondents in this study recognized that SDM was usually employed during their most recent doctor's appointment. Perceived experience of DM was higher among respondents with better health and higher health literacy, as well as those who felt the appointment was sufficiently long for discussion. Conversely, elements of SDM were reported less frequently by respondents with lower educational attainment and those who felt that the diagnosis contradicted their own impressions or information they had found online. The findings were consistent regardless of respondent's age or gender. We conclude that the quality of clinical DM can be improved by allocating sufficient time for meaningful discussions and tailoring communication to the patient's level of health literacy.

## Supplemental Material

sj-docx-1-jpx-10.1177_23743735251415086 - Supplemental material for Experiences of Decision-Making in Healthcare and Online Health Information-Seeking Among Older Adults and People with Long-Term Disease: Online Survey Study Supplemental material, sj-docx-1-jpx-10.1177_23743735251415086 for Experiences of Decision-Making in Healthcare and Online Health Information-Seeking Among Older Adults and People with Long-Term Disease: Online Survey Study by Milla Rosenlund, Tuuli Turja, Virpi Jylhä, Kaija Saranto and Hanna Kuusisto in Journal of Patient Experience

sj-docx-2-jpx-10.1177_23743735251415086 - Supplemental material for Experiences of Decision-Making in Healthcare and Online Health Information-Seeking Among Older Adults and People with Long-Term Disease: Online Survey Study Supplemental material, sj-docx-2-jpx-10.1177_23743735251415086 for Experiences of Decision-Making in Healthcare and Online Health Information-Seeking Among Older Adults and People with Long-Term Disease: Online Survey Study by Milla Rosenlund, Tuuli Turja, Virpi Jylhä, Kaija Saranto and Hanna Kuusisto in Journal of Patient Experience

sj-docx-3-jpx-10.1177_23743735251415086 - Supplemental material for Experiences of Decision-Making in Healthcare and Online Health Information-Seeking Among Older Adults and People with Long-Term Disease: Online Survey Study Supplemental material, sj-docx-3-jpx-10.1177_23743735251415086 for Experiences of Decision-Making in Healthcare and Online Health Information-Seeking Among Older Adults and People with Long-Term Disease: Online Survey Study by Milla Rosenlund, Tuuli Turja, Virpi Jylhä, Kaija Saranto and Hanna Kuusisto in Journal of Patient Experience

sj-docx-4-jpx-10.1177_23743735251415086 - Supplemental material for Experiences of Decision-Making in Healthcare and Online Health Information-Seeking Among Older Adults and People with Long-Term Disease: Online Survey Study Supplemental material, sj-docx-4-jpx-10.1177_23743735251415086 for Experiences of Decision-Making in Healthcare and Online Health Information-Seeking Among Older Adults and People with Long-Term Disease: Online Survey Study by Milla Rosenlund, Tuuli Turja, Virpi Jylhä, Kaija Saranto and Hanna Kuusisto in Journal of Patient Experience

## Abbreviations

ANOVA: analysis of variance
CFA: confirmatory factor analysis
DM: decision-making
HCP: healthcare professional
MS: multiple sclerosis
SD: standard deviation
SDM: shared decision-making
SDM-Q-9: the 9-Item Shared Decision-Making Questionnaire
