# Supplementary material for: Experiences of Decision-Making in Healthcare and Online Health Information-Seeking Among Older Adults and People with Long-Term Disease: Online Survey Study
Source: J Patient Exp. 2026 Jan 20;13:23743735251415086. doi: 10.1177/23743735251415086 (PMC12819975; doi:10.1177/23743735251415086)
Supplement: sj-docx-1-jpx-10.1177_23743735251415086 - Supplemental material for Experiences of Decision-Making in Healthcare and Online Health Information-Seeking Among Older Adults and People with Long-Term Disease: Online Survey Study [file sj-docx-1-jpx-10.1177_23743735251415086.docx]

Appendix 1. Survey items

| Item | Response options |
| --- | --- |
| *Sociodemographic factors and subjective health status* | |
| Gender | Female |
|  | Male |
|  | Other |
| Age | (report your age in years) _______ |
| Your highest education level | Elementary school or similar |
|  | High school or vocational education |
|  | Bachelor’s degree |
|  | Master’s degree |
|  | Doctoral or licentiate degree |
| How do you feel about your current health? | Good |
|  | Fairly good |
|  | Average |
|  | Fairly poor |
|  | Poor |
| *Characteristics of the appointment* | |
| Have you had an appointment during the past six weeks*?* | Yes  No |
| The main reason for the appointment was (choose one alternative) | Long-term illness (e.g. medication, follow-up)  Diagnosing the symptom/disease  Preventive care  Other reason |
| The purpose of the appointment was | First appointment |
|  | Follow-up |
|  | Periodic follow-up for long-term illness |
|  | First appointment |
| Did you visit at | General practitioner |
|  | Specialist |
|  | Occupational physician |
| Location of the appointment was | Face to face |
|  | Phone call |
|  | Video or other distance connection |
| The appointment duration was sufficiently long enough to discuss all the relevant issues | Completely disagree  Partly disagree  Partly agree  Completely agree |
| The diagnosis or treatment decision made by the doctor contradicted the information I had found beforehand on the Internet | Completely disagree  Partly disagree  Partly agree  Completely agree  I did not search the internet for information beforehand |
| The diagnosis or treatment decision I received contradicted my own feelings | Completely disagree  Partly disagree  Partly agree  Completely agree |
| *Managing the health information* | |
| How often do you search for information concerning your own health? | Not at all or a few times a year  Once or a few times a month  Once or few times a week  Daily |
|  |  |
| I have enough information to participate in a discussion about my health | Completely disagree  Partly disagree  Partly agree  Completely agree |
|  |  |
| I understand what the findings concerning my treatment mean | Completely disagree  Partly disagree  Partly agree  Completely agree |
