# Supplementary material for: Experiences of Decision-Making in Healthcare and Online Health Information-Seeking Among Older Adults and People with Long-Term Disease: Online Survey Study
Source: J Patient Exp. 2026 Jan 20;13:23743735251415086. doi: 10.1177/23743735251415086 (PMC12819975; doi:10.1177/23743735251415086)
Supplement: sj-docx-2-jpx-10.1177_23743735251415086 - Supplemental material for Experiences of Decision-Making in Healthcare and Online Health Information-Seeking Among Older Adults and People with Long-Term Disease: Online Survey Study [file sj-docx-2-jpx-10.1177_23743735251415086.docx]

Appendix 2. SDM-Q-9-FIN survey items

| SDM-Q-9-FIN item (translated in English) | Response options |
| --- | --- |
| The doctor made clear that a decision needs to be made.  The doctor wanted to know exactly how I want to be involved in making the decision.  The doctor told me that there are different options for treating my medical condition.  The doctor precisely explained the advantages and disadvantages of the treatment options.  The doctor helped me understand all the information.  The doctor and I selected a treatment option together.  The doctor asked me which treatment option I prefer.  The doctor and I thoroughly weighed the different treatment options.  The doctor made clear that a decision needs to be made. | Completely disagree  Partly disagree  Partly agree  Completely agree |
