# Supplementary material for: Experiences of Decision-Making in Healthcare and Online Health Information-Seeking Among Older Adults and People with Long-Term Disease: Online Survey Study
Source: J Patient Exp. 2026 Jan 20;13:23743735251415086. doi: 10.1177/23743735251415086 (PMC12819975; doi:10.1177/23743735251415086)
Supplement: sj-docx-4-jpx-10.1177_23743735251415086 - Supplemental material for Experiences of Decision-Making in Healthcare and Online Health Information-Seeking Among Older Adults and People with Long-Term Disease: Online Survey Study [file sj-docx-4-jpx-10.1177_23743735251415086.docx]

Appendix 4. SDM-Q-9-FIN single item mean scores and total scores in respondent groups

|  | **Total** | **Finnish Pensioners’ Federation (n=629)** | **Patient associations (n=107)** | **Women (n=472)** | **Men (n=264)** |
| --- | --- | --- | --- | --- | --- |
| The doctor made clear that a decision needs to be made. | 3.38 | 3.40 | 3.47 | 3.35 | 3.42 |
| The doctor wanted to know exactly how I want to be involved in making the decision. | 2.80 | 2.85 | 2.71 | 2.78 | 2.84 |
| The doctor told me that there are different options for treating my medical condition. | 2.75 | 2.83 | 2.28 | 2.74 | 2.78 |
| The doctor precisely explained the advantages and disadvantages of the treatment options. | 2.72 | 2.79 | 2.55 | 2.68 | 2.78 |
| The doctor helped me understand all the information. | 3.18 | 3.23 | 3.06 | 3.17 | 3.20 |
| The doctor and I selected a treatment option together. | 2.90 | 2.94 | 2.74 | 2.89 | 2.94 |
| The doctor asked me which treatment option I prefer. | 2.44 | 2.47 | 2.19 | 2.42 | 2.47 |
| The doctor and I thoroughly weighed the different treatment options. | 2.51 | 2.57 | 2.21 | 2.50 | 2.53 |
| The doctor and I reached an agreement on how to proceed. | 3.28 | 3.31 | 3.12 | 3.26 | 3.32 |
| Total (mean) | 2.88 | 2.93 | 2.76 | 2.80 | 2.92 |
